# Treatment Practices, Outcomes, and Costs of Multidrug-Resistant and Extensively Drug-Resistant Tuberculosis, United States, 2005–2007

**DOI:** 10.3201/eid2005.131037

**Published:** 2014-05

**Authors:** Suzanne M. Marks, Jennifer Flood, Barbara Seaworth, Yael Hirsch-Moverman, Lori Armstrong, Sundari Mase, Katya Salcedo, Peter Oh, Edward A. Graviss, Paul W. Colson, Lisa Armitige, Manuel Revuelta, Kathryn Sheeran, the TB Epidemiologic Studies Consortium

**Affiliations:** Centers for Disease Control and Prevention, Atlanta, Georgia, USA (S.M. Marks, L. Armstrong, S. Mase, TB Epidemiologic Studies Consortium);; California Department of Public Health, Richmond, California, USA (J. Flood, K. Salcedo, P. Oh);; Texas Department of State Health Services, Tyler, Texas, USA (B. Seaworth, L. Armitige, K. Sheeran);; University of Texas Health Science Center, Tyler (B. Seaworth, L. Armitige, K. Sheeran);; ICAP/Columbia University, New York, New York, USA (Y. Hirsch-Moverman, P.W. Colson, M. Revuelta);; Methodist Hospital Research Institute, Houston, Texas, USA (E.A. Graviss)

**Keywords:** Tuberculosis, drug resistance, cost, treatment practices, outcomes, multidrug-resistant tuberculosis, extensively drug-resistant tuberculosis, TB, tuberculosis and other mycobacteria, United States

## Abstract

To describe factors associated with multidrug-resistant (MDR), including extensively-drug-resistant (XDR), tuberculosis (TB) in the United States, we abstracted inpatient, laboratory, and public health clinic records of a sample of MDR TB patients reported to the Centers for Disease Control and Prevention from California, New York City, and Texas during 2005–2007. At initial diagnosis, MDR TB was detected in 94% of 130 MDR TB patients and XDR TB in 80% of 5 XDR TB patients. Mutually exclusive resistance was 4% XDR, 17% pre-XDR, 24% total first-line resistance, 43% isoniazid/rifampin/rifabutin-plus-other resistance, and 13% isoniazid/rifampin/rifabutin-only resistance. Nearly three-quarters of patients were hospitalized, 78% completed treatment, and 9% died during treatment. Direct costs, mostly covered by the public sector, averaged $134,000 per MDR TB and $430,000 per XDR TB patient; in comparison, estimated cost per non-MDR TB patient is $17,000. Drug resistance was extensive, care was complex, treatment completion rates were high, and treatment was expensive.

## Abstract 2

Drug resistance was extensive and care was complex; nevertheless, high rates of treatment completion were achieved albeit at considerable cost.

Drug-resistant *Mycobacterium tuberculosis* poses substantial obstacles to tuberculosis (TB) control. In the United States, multidrug-resistant (MDR) TB (resistant to at least isoniazid and rifampin) comprises only 1.0%–1.5% of TB cases but requires lengthy regimens of toxic drugs, imposes high costs on the health care system and society, and causes high mortality rates.

Studies of MDR TB in the United States have been limited by small sample sizes, limited study periods, minimal information on outcomes and costs, or reliance solely on surveillance data (*1*–*6*), which omit some cases of acquired drug resistance and changes in regimens. Costs of treating MDR TB are not routinely collected or reported.

Our study describes and analyzes characteristics associated with drug resistance, timely diagnosis, treatment practices, outcomes, and costs associated with MDR TB for cases reported to the Centers for Disease Control and Prevention (CDC) by California, New York, NY, and Texas during 2005–2007. These 3 areas contribute about half of US MDR TB cases annually.

## Methods

CDC and local institutional review boards approved the study and granted a waiver of patient informed consent and patient authorization. We defined a 5-drug regimen (Technical Appendix) to be consistent with US and World Health Organization recommendations (7,8). All study definitions are in the Technical Appendix.

Each site identified cases of MDR TB and extensively drug-resistant (XDR) TB reported to CDC during 2005–2007. The study included all XDR TB cases and a 75% simple random sample of MDR TB cases from California and New York, NY (New York City), and a 50% sample from Texas. Using standardized forms, we abstracted hospital, laboratory, and public health clinic records retrospectively for patient demographic, socioeconomic, and clinical characteristics and for treatment, case management, outcomes, and costs. Total charges for each TB-associated hospitalization were abstracted from hospital UB-04 forms. To ascertain sputum-culture conversion and drug resistance, we examined all available culture and drug-susceptibility testing (DST) results from diagnosis through treatment.

To assess representativeness, we compared our sample with National TB Surveillance System data from all US sites. We identified characteristics among MDR TB patients associated with the following 3 dichotomous outcomes: drug resistance acquisition, expert consultation use, and death during TB treatment. Multivariable logistic regression was used with backward selection at p<0.05 to identify variables remaining in final models (SAS version 9.2/9.3; SAS Institute, Cary, NC, USA). Adjusted odds ratios (AORs) significant at 95% CIs and Schwarz Criterion statistics are reported for goodness-of-fit. Variables included in initial models were patient demographics (gender, age group, race/ethnicity, foreign birth), socioeconomic factors (homelessness, unemployment, illicit substance use, excess alcohol use, smoking), medical risks (HIV infection, diabetes), TB history, disease severity (acid-fast bacilli [AFB]–smear positivity, cavitation, dissemination), drug-resistance pattern, receipt of TB clinic outpatient care, and additional relevant characteristics (for acquired resistance: receipt of ≥4 effective medications; for expert consultation: incarceration in a correctional institution, long-term-care facility residence, pregnancy, death during treatment, number of adverse events, private outpatient insurance; for death during treatment: pregnancy, incarceration in a correctional institution).

Inpatient costs were measured; outpatient costs and productivity losses were estimated (Technical Appendix). Hospital charges were converted to costs by using hospital-specific operating cost-to-charge ratios (*9*). All costs were converted to 2010 US dollars (*10*) and were adjusted for cost of living (1.13 for California, 1.08 for New York City, and 0.94 for Texas) (*11*) to facilitate aggregation. For 17 patients for whom hospital charges data were missing, we multiplied hospitalization duration by average cost per day for patients for whom data were available ($1,419).

Study total direct costs were compared with estimated direct costs for cases of non-MDR TB (Technical Appendix). We report SEMs to display cost variability.

For productivity losses from hospitalization, we applied an updated 2010 dollar value of work-plus-home production of $224/day for employed patients and $40/day for home-only production of unemployed patients (*12*). For TB-related deaths, we estimated the value of remaining lifetime productivity, updated to 2010 dollars, based on the age at death (*12*). For patients experiencing adverse events during treatment, we calculated a disability adjustment per patient (100%, 83%, 67%, 50%, 33%, 17%, 0%). We estimated direct and productivity-loss costs and examined associated characteristics by using multivariable linear regression with backward selection. Adjusted R^2^ statistics are reported to show goodness-of-model-fit.

## Results

The sample consisted of 135 patients (130 with MDR TB and 5 with XDRTB), representing 36% (130/364) of MDR TB and 56% (5/9) of XDR TB cases reported in the United States during 2005–2007. Among these patients, 87% were foreign born and 36% had prior TB disease (Tables 1–3). Among patients for whom information about concurrent medical conditions was available, 24 (20%) of 121 had diabetes and 14 (12%) of 116 had HIV infection. The study population resembled all US MDR TB patients; however, fewer study participants were White or unemployed, and more used noninjection drugs, had prior TB, or had AFB-positive smear specimens. Similar to all foreign-born TB patients in the United States, most foreign-born MDR TB study participants arrived from Mexico, the Philippines, India, and Vietnam. Of the 135 patients, 7% were homeless before diagnosis (6 patients) or during treatment (3 patients). Case management to obtain housing during treatment was needed by 23 (17%) patients; 38 (28%) patients had been unemployed before diagnosis, and of the 97 remaining patients, 27% stopped work because of MDR TB.

**Table 1:** Demographic characteristics of study participants and all patients with MDR and/or XDR TB, United States, 2005–2007*

Characteristic	Study participants, no. (%), n = 135	All US MDR TB patients, no. (%), n = 370
Sex		
M	68 (50)	205 (55)
F	67 (50)	164 (44)
Age, y		
Median	38.2	
0–14	1 (1)	13 (4)
15–24	24 (18)	64 (17)
25–44	62 (46)	171 (46)
45–64	40 (30)	91 (25)
>65	8 (6)	31 (8)
Race/ethnicity		
Hispanic	42 (31)	100 (27)
White†	4 (3)	31 (8)
Black	14 (10)	63 (17)
Asian	72 (53)	173 (47)
Other/unknown	3 (2)	3 (1)
Geographic origin		
Foreign born‡	118 (87)	305 (82)
US born	17 (13)	64 (17)

*Study patients were from California, New York City, and Texas. MDR, multiple-drug resistant; XDR, extensively drug resistant; TB, tuberculosis. †Statistically significant differences between percentages of study patients and all US MDR TB patients at p<0.05. ‡For 110 participants, median no. years after first entry into United States = 3.5.

**Table 3:** Clinical characteristics of study participants and other patients with MDR and/or XDR TB, United States, 2005–2007*

Characteristic	Study participants, no. (%), n = 135	All US MDR TB patients, no. (%), n = 370
History of LTBI, % of 130 known	21 (16)	
History of completing LTBI Rx, % of 21 with history of LTBI	14 (67)	
History of TB disease†	48 (36)	60 (16)
Contact with infectious TB patient, % of 87 known	10 (11)	
Contact with infectious MDR TB patient, % of 10 contacts	6 (60)	
Dead at TB diagnosis	1 (1)	5 (1)
Smear positive at any time, % of 134 alive at diagnosis†	103 (77)	223 (60)
Sites of TB disease		
Pulmonary	115 (85)	332 (90)
Extrapulmonary only	8 (6)	37 (10)
Disseminated at any time	12 (9)	
Extent of pulmonary disease at diagnosis		
Extensive	69	
Moderate	31	
Minimal	21	
Undocumented	6	
Description of extensive pulmonary disease, at any time		
Miliary	4	
Cavitary	58	127
Multiple lobes	70	
Collapsed lobes	6	
Extent of extrapulmonary disease at diagnosis		
Extensive	2	
Moderate	3	
Minimal	3	
*MDR, multidrug resistant; XDR, extensively drug resistant; TB, tuberculosis; LTBI, latent TB infection; Rx, treatment. Blank cells indicate data not available. †Statistically significant differences between percentages of study patients and all US MDR TB patients at p<0.05.		

Of the 135 patients, disease was pulmonary for 85%, extrapulmonary only for 6%, and disseminated for 9%. Of 127 patients with pulmonary or disseminated disease, 69 (54%) had extensive disease. Of 8 patients with extrapulmonary-only disease, 2 (25%) had extensive disease. Of 134 patients alive at diagnosis, 77% (103) had at least 1 AFB-smear–positive specimen, 72% from sputum.

### Drug Resistance

**Figure 1:**
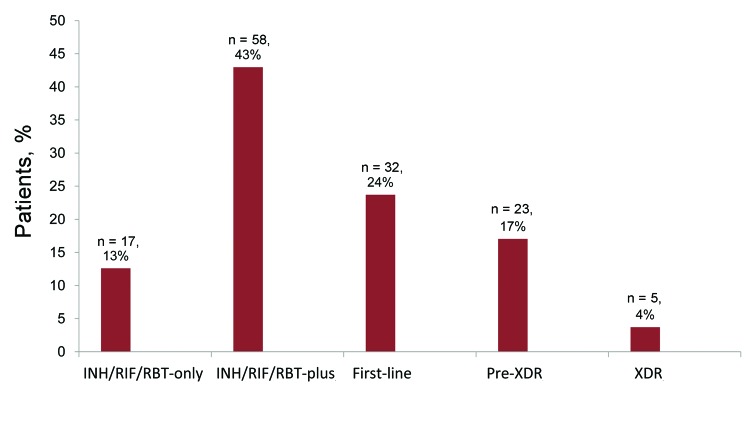
Percentage of 135 patients for whom *Mycobacterium tuberculosis* isolates had the following mutually exclusive resistance patterns. INH/RIF/RBT-only, resistant to isoniazid (INH)/rifampin (RIF)/rifabutin (RBT) only; INH/RIF/RBT-plus, resistant to a median of 4 medications; first-line, resistant to a median of 6 medications; pre-XDR, resistant to a median of 8 medications; XDR, resistant to a median of 11 medications.

At various times during treatment, *M. tuberculosis* isolates were tested for susceptibility (median 14 medications, range 4–19) and were resistant to several (median 5 medications, range 2–16) at any time during treatment. The following mutually exclusive resistance patterns were identified: 4% XDR, 17% pre-XDR, 24% total first-line resistance, 43% isoniazid/rifampin/rifabutin-plus-other resistance, and 13% isoniazid/rifampin/rifabutin-only resistance (Figure 1). Initial *M. tuberculosis* isolates obtained within 30 days of treatment initiation revealed isoniazid/rifampin resistance among 122 (94%) of the 130 MDR TB patients and XDR among 4 (80%) of the 5 XDR TB patients. DST was conducted for first-line drugs during the first month of treatment, for second-line drugs during the second month of treatment, for linezolid during the fourth month, and for clofazimine during the seventh month.

Among 128 patients for whom DST was conducted multiple times on separate dates, acquired resistance to an anti-TB medication during treatment was detected for 27 (21%). According to multivariable analysis, acquisition of drug resistance during treatment was more likely for patients who were recently homeless (AOR 5.8, 95% CI 1.2–28.9), who had pre-XDR or XDR TB (AOR 5.1, 95% CI 1.9–14.2), or who were Black (AOR 4.1, 95% CI 1.1–15.4) (Table 4). Acquisition of resistance to isoniazid or rifampin, resulting in MDR TB, occurred for 6% of patients, to fluoroquinolones for 3%, and to injectable drugs for 4%. Other medications to which resistance was acquired were ethambutol (10% of patients), pyrazinamide (6%), streptomycin (5%), ethionamide (5%), rifabutin (2%), cycloserine (1%), and clofazimine (1%). For 1 patient, fluoroquinolone resistance was acquired, resulting in XDR TB.

**Table 4:** Characteristics associated with any acquired antimicrobial drug resistance during MDR TB Treatment, 128 patients, California, Texas, and New York City, USA, 2005–2007*†

Variable	Initial OR estimate	Initial 95% CI	Initial Pr>χ^2^	Final OR estimate	Final 95% CI	Final Pr>χ^2^
Age >65 y	<0.001	<0.001–>999.999	0.973			
**Black race**	33.19	0.80–>999.999	0.065	**4.07**	**1.08–15.37**	**0.039**
**Recent homelessness**	18.76	0.93–377.71	0.056	**5.81**	**1.17–28.86**	**0.031**
**Pre-XDR or XDR TB**	8.78	2.31–33.42	0.001	**5.15**	**1.86–14.21**	**0.002**
AFB-smear positive	5.34	0.86–33.22	0.072			
Age 25-44 y	4.83	0.65–36.03	0.124			
Hispanic ethnicity	4.83	0.11–216.20	0.417			
≥4 Effective medications	3.46	0.41–29.47	0.256			
Age 45–64 y	3.41	0.39–30.21	0.271			
Asian race	3.35	0.07–151.32	0.534			
Disseminated TB disease	2.28	0.21–24.20	0.495			
Foreign born	2.10	0.15–28.64	0.577			
Recent cigarette smoker	1.96	0.41–9.31	0.397			
Recent excess alcohol use	0.99	0.13–7.65	0.988			
TB clinic outpatient management	0.98	0.18–5.35	0.980			
Recent unemployment	0.97	0.24–3.93	0.964			
Cavitary disease	0.77	0.21–2.84	0.694			
Diabetes	0.76	0.16–3.70	0.732			
History of TB disease	0.44	0.12–1.59	0.208			
HIV infection	0.39	0.03–4.92	0.463			
Recent injection drug or noninjection drug use	0.15	0.00–5.08	0.291			

*MDR TB, multidrug-resistant tuberculosis; XDR TB, extensively drug-resistant TB; OR, odds ratio; AFB, acid-fast bacilli; Pr, probability. Boldface indicates significance in the final model. Blank cells indicate variables not retained in the final model. †Model fit intercept and covariates: Akaike information criterion, initial 136.392, final 116.619; Schwarz criterion, initial 198.964, final 127.996; –2logL, initial 92.392, final 108.619.

### Time to Diagnosis

For 74 patients who had TB symptoms before diagnosis, a median of 1.6 months elapsed from symptom onset to initial TB diagnosis. Of 134 patients alive at diagnosis, 123 (92%) started receiving ≥2 second- or third-line medications a median of 2.4 months after initial TB diagnosis. The median duration of infectiousness was 10 months.

### Treatment Practices

Among 105 patients who completed treatment, treatment duration varied. Median durations were 32.3 months (interquartile range [IQR] 30.6–37.8) for those with XDR, 25.1 months (IQR 23.6–29.2) for those with pre-XDR, 25.7 months (IQR 22.4–26.9) for those with total-first-line–resistant, 24.1 months (IQR 20.1–27.0) for those with isoniazid/rifampin/rifabutin-plus–resistant, and 20.0 months (IQR 19.4–24.5) for those with isoniazid/rifampin/rifabutin-only–resistant TB.

Providers changed medications for 134 patients alive at diagnosis a median of 7 times during treatment; 33% of 988 medication changes were because of adverse events and 10% because of DST results. Of 134 patients, 34% received a 5-drug regimen before sputum-culture conversion, and 61% ultimately received a 5-drug regimen. Of the 134 patients, 81% received an effective (i.e., medication to which their isolate was never resistant) injectable medication and 86% received an effective fluoroquinolone medication during treatment. Of 123 patients who received outpatient care, 90% received >80% of outpatient medication doses by directly observed therapy (DOT).

Most study patients were hospitalized for TB, often several times; 73% (98) were hospitalized at least 1 time and 29% were hospitalized >2 times (range 2–6). Detailed data were available for 83% of inpatients. Among multiple possible reasons, severe worsening of TB disease was the reason for 50% of hospitalizations, followed by the need to initiate or change treatment (40%), implement respiratory isolation (21%), manage adverse events (7%), manage concurrent conditions (3%), and perform surgery (1%). One patient had undergone TB-related lung lobectomy. Four XDR TB patients were hospitalized for a median of 282 days (range 14–850) and non-XDR patients for a median of 27 days (range 1–759). Home isolation was prescribed for 37% of patients; a median of 102 days (range 4–337) for non-XDR TB patients and 257 days for the 1 XDR TB patient.

Of the 134 patients alive at diagnosis, 81% had documentation of physician consultation with an MDR TB expert during inpatient or outpatient care. Expert consultation was more likely for patients managed primarily by a TB clinic (AOR 5.7, 95% CI 1.9–16.8) and less likely for those with private insurance (AOR 0.2, 95% CI = 0.1–0.7) (Table 5). Overall, each patient received a median of 3 expert consultations.

**Table 5:** Characteristics associated with expert consultation for 134 patients during MDR TB treatment, California, Texas, and New York, NY, USA, 2005–2007*†

Variable	Initial OR estimate	Initial 95% CI	Initial Pr>χ^2^	Final OR estimate	Final 95% CI	Final Pr>χ^2^
XDR TB	>999.999	<0.001–>999.999	0.939			
Recent homelessness	>999.999	<0.001–>999.999	0.887			
Correctional institution residence	>999.999	<0.001–>999.999	0.939			
Recent injection drug or noninjection drug use	61.63	0.00–>999.999	0.401			
Recent cigarette smoker	27.88	0.56–>999.999	0.096			
Diabetes	26.44	1.28–545.80	0.034			
Disseminated TB disease	9.49	0.18–501.11	0.266			
**TB clinic outpatient management**	7.96	1.50–42.32	0.015	**5.67**	**1.93–16.64**	**0.002**
Age 45–64 y	4.30	0.38–48.55	0.239			
Recent unemployment	3.81	0.52–28.18	0.190			
Age 25–44 years	2.92	0.58–14.63	0.192			
Long-term care facility resident	1.87	<0.001–>999.999	0.878			
5-drug regimen	1.13	0.22–5.82	0.882			
Total no. adverse events	1.12	0.88–1.42	0.371			
Total first-line resistance	0.63	0.11–3.77	0.614			
Acquired resistance	0.57	0.07–4.37	0.588			
Foreign born	0.55	0.03–12.28	0.705			
History of TB disease	0.53	0.13–2.10	0.363			
Age >65 y	0.50	0.02–14.97	0.687			
HIV infection	0.34	0.02–7.21	0.492			
Pregnant	0.28	0.02–5.15	0.393			
**Private insurance**	0.14	0.02–0.87	0.035	**0.23**	**0.08–0.68**	**0.008**
Pre-XDR	0.12	0.01–1.35	0.087			
Male	0.12	0.02–0.82	0.031			
Died	0.05	<0.001–3.46	0.165			
White race	0.05	<0.001–>999.999	0.988			
**Recent excess alcohol use**	<0.001	<0.001–0.06	0.002	**0.19**	**0.05–0.72**	**0.014**
Black race	<0.001	<0.001–>999.999	0.937			
Hispanic ethnicity	<0.001	<0.001–>999.999	0.938			
Asian race	<0.001	<0.001–>999.999	0.943			

*MDR TB, multidrug-resistant tuberculosis; XDR TB, extensively drug-resistant TB; OR, odds ratio. Boldface indicates significance in the final model. Blank cells indicate variables not retained in the final model. †Model fit intercept and covariates: AIC (initial 133.402, final 121.683), SC (initial 223.235, final 133.274**); –**2logL (initial 71.402, final 113.683).

Of the 134 patients alive at diagnosis, ≈90% were assigned a case manager. Case management activities included home visits (68%), social worker assistance (37%), transportation assistance (32%), incentives (25%), housing assistance (17%), and other activities including legal orders for DOT or isolation (9%–15%). Only 4% of patients received none of these case management services. Interpreter use was documented for 60% of 107 patients who understood some or no English.

### Outcomes

Of 112 eligible patients, including all XDR TB patients, sputum culture converted to negative for 109 (97%). Patients considered ineligible for culture conversion included 1 patient whose TB was diagnosed after death, 6 who died during treatment, 3 who were transferred to another US jurisdiction or out of the United States, 7 who had extrapulmonary-only disease, and 6 without a positive sputum culture result. Of the 3 for whom no culture conversion was documented, 1 was lost to follow-up after 166 days and 2 completed treatment. Culture conversion occurred within a median of 2 months from starting a 5-drug-regimen but varied by resistance pattern (Technical Appendix).

Of the 134 patients alive at diagnosis, 78% completed treatment, 11% transferred within or outside the United States or were lost to follow-up, and 1% stopped treatment because of adverse events (Table 6). For no patients did treatment fail or TB recur within the year after treatment completion. Of the 134 patients, 12 (9%) died during treatment; 75% of these deaths were considered TB related. No XDR TB patient died. Death during treatment was significantly associated with age >65 years (AOR 20.2, 95% CI 2.3–181.0), smoking (AOR 6.4, 95% CI 1.0–39.4), or HIV infection (AOR 6.3, 95% CI 1.1–37.7) (Technical Appendix). When TB medications and interaction terms were initially included in the model, HIV infection was no longer associated with death and receipt of an effective injectable medication was associated with lower odds of dying (AOR 0.02, 95% CI 0.002–0.2) (Technical Appendix). No HIV-infected patient who received an effective injectable medication died. Of 9 patients who died of TB-related causes, only 2 who had received a 5-drug regimen died, both after 8 months; 1 had received 3 non–first-line medications and died after 5 months, and the remaining 6 never received >2 MDR TB second- or third-line medications and died within 49 days of treatment initiation.

**Table 6:** Treatment outcomes of MDR/XDR TB study patients alive at diagnosis, by resistance pattern, California, Texas, and New York City, USA, 2005–2007*

Resistance pattern	Completed treatment, %	Transferred within United States, %	Transferred out of United States, %	Lost to follow-up, %	Stopped because of side effects, %	Died during treatment, %
All, N = 134	78	3	6	2	1	9
INH/RIF/RBT-only, n = 17	59	0	18	6	0	18
INH/RIF/RBT-plus, n = 58	83	3	7	0	0	7
First-line, n = 32	78	0	3	6	3	9
Pre-XDR, n = 22	77	9	0	0	5	9
XDR, n = 5	100	0	0	0	0	0
*MDR TB, multidrug-resistant tuberculosis; XDR TB, extensively drug-resistant TB; INH, isoniazid; RIF, rifampin; RBT, rifabutin.						

Of the 4 HIV-infected patients who died of TB-related causes, 3 had a CD4 count of <50 cells/mm^3^ at initiation of TB treatment and the other had a CD4 count of <100 cells/mm^3^. Of these 4 patients, 2 were taking >3 anti-HIV medications.

Among patients alive at diagnosis, a median of 1 adverse event (average 2.9) resulted in medication change or adjustment, but only 2 patients completely stopped treatment. According to multivariate linear regression, use of clofazimine was significantly associated (p<0.05) with more adverse events but was not associated with death during treatment. Of 9 patients receiving clofazimine, 6 experienced postinitiation gastrointestinal effects that resulted in their discontinuing clofazimine. Because of MDR TB or its treatment, of the 134 patients, 13% experienced hearing impairment, 13% hepatitis, 11% renal impairment, 8% difficulty ambulating, 7% visual impairment, and 1% seizures. Depression or psychosis was documented for 19% (80% of whom were taking cycloserine), and pulmonary impairment was documented for 4%. Of 103 impairments, 66% were mild, but another 7% were graded most severe and occurred for 6 patients, 3 of whom completed treatment; 2 of those patients died (lung and mobility impairments), and 1 transferred outside the country. 

### Cost of MDR TB and XDR TB

**Figure 2:**
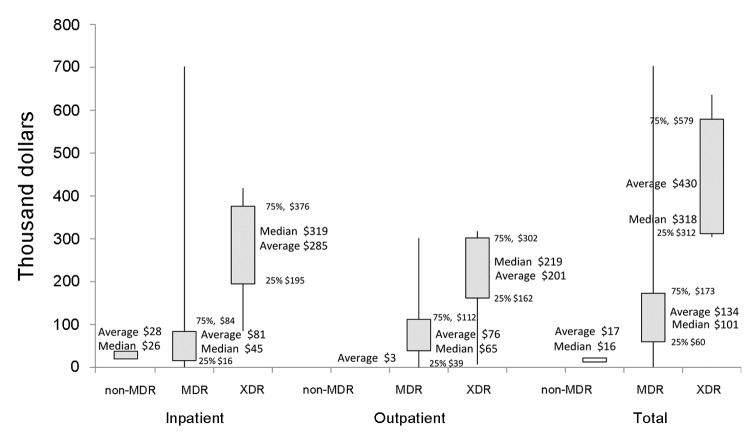
Average, median, and distribution of direct costs per patient in 2010 US dollars by drug resistance. This box-plot diagram shows the minimum and maximum values (vertical lines), the averages and medians (numbers), and the interquartile ranges (box). MDR, multidrug-resistant tuberculosis; XDR, extensively drug-resistant tuberculosis.

Direct costs averaged $134,000 (SE $9,683) per MDR TB patient and $430,000 (SE $73,109) per XDR TB patient. In comparison, costs are estimated at $17,000 (SE $1,210) per non-MDR TB patient (Figure 2 and Technical Appendix). For isoniazid/rifampin/rifabutin-only, direct costs averaged $77,000 (SE $15,448) and direct-plus-productivity-loss costs averaged $226,000 (SE $73,338). Outpatient medications comprised ≈40% of direct costs, averaging $53,300 for MDR TB and $164,000 for XDR TB patients. Direct-plus-productivity-loss costs averaged $260,000 (SE $23,212) per MDR TB patient and $554,000 (SE = $127,707) per XDR TB patient. Highest costs were nearly $1.8 million. Applying these averages to 364 cases of MDR TB and 9 cases of XDR TB in the United States during 2005–2007, direct costs were ≈$53 million and direct-plus-productivity-loss costs were ≈$100 million.

When days hospitalized were controlled for, characteristics associated with greater direct costs, in descending order, were having XDR TB, residency in a long-term-care institution, non–injection-drug use, HIV infection, or having public insurance (adjusted R^2^ = 0.55). When days hospitalized were controlled for, characteristics associated with greater direct-plus-productivity-loss costs were death, XDR TB, non–injection-drug use, HIV-infection, diabetes, or being male (R^2^ = 0.66) (Technical Appendix)

Table 2 shows that known insurance status was indicated on clinic records of 112 patients. Of 76 patients for whom insurance coverage while hospitalized was known, 38% had public insurance (including 32% Medicaid), 36% had no insurance, 24% had private insurance, 4% were in jail/prison, and 1% had other insurance. The public sector covered (i.e., by public insurance, for outpatients who had no/unknown insurance, for uninsured inpatients cared for in publicly financed hospitals, for jail/prison inmates) 75% of MDR TB and 100% of XDR TB patients and incurred 80% of MDR TB direct costs ($13,883,000/$17,415,000) and 100% of XDR TB direct costs ($2,149,000).

**Table 2:** Socioeconomic characteristics of study participants and all patients with MDR and/or XDR TB, United States, 2005–2007*

Characteristic	Study participants, no. (%), n = 135	All US MDR TB patients, no. (%), n = 370
Unemployed†	38 (28)	187 (51)
Homeless	9 (7)	20 (5)
Correctional institution resident	6 (4)	7 (2)
Long-term care facility resident	4 (3)	6 (2)
Injection drug use	5 (4)	8 (2)
Noninjection drug use†	12 (9)	15 (4)
Excess alcohol use	15 (11)	31 (8)
Smoker	31 (23)	
Pregnant at treatment initiation	6 (4)	
Private health insurance, % of 112 known	24 (21)	
Public health insurance, % of 112 known	49 (44)	
Jail/prison health coverage, % of 112 known	2 (2)	
Other health insurance, % of 112 known	5 (4)	
No health insurance, % of 112 known	32 (29)	
HIV+	14 (10)	29 (8)
Receiving HAART	9 (64)	
Receiving ART	1 (7)	
Receiving neither HAART nor ART	3 (21)	
Receipt of ART not documented	1 (7)	
Not HIV infected	102 (76)	205 (55)
HIV status unknown	19 (14)	136 (37)
Diabetes, % of 121 known	24 (20)	
ESRD, % of 121 known	3 (2)	
Prolonged corticosteroid therapy, % of 121 known	2 (2)	
Other immunosuppressive therapy, % of 121 known	2 (2)	
Cancer, % of 121 known	3 (2)	
Hematologic diseases, % of 121 known	2 (2)	

*Study patients were from California, New York City, and Texas. MDR, multidrug resistant; XDR, extensively drug resistant; TB, tuberculosis; HIV+, HIV infected; HAART, highly active antiretroviral therapy; ART, antiretroviral therapy; ESRD, end-stage renal disease. Blank cells indicate data not available. †Statistically significant differences between percentages of study patients and all US MDR TB patients at p<0.05.

## Discussion

In this population-based sample, which comprised 36% (130/364) of MDR TB and 56% (5/9) of XDR TB cases reported in the United States during 2005–2007, MDR/XDR TB diagnosis and treatment were very complex: *M. tuberculosis* isolates were resistant to a large number of medications, care was complicated by extensive disease and by concurrent conditions, and patients were highly infectious.

Despite this complexity, for nearly all eligible patients, sputum cultures converted to negative and 78% of patients completed treatment, including all those with XDR TB. Only 1% stopped treatment because of adverse events. The mortality rate (10%) was lower than that for other countries (*13**–**17*), and the mortality rate for patients during treatment (9%) was similar to that for US patients with isoniazid/rifampin-susceptible TB (8%) (L. Armstrong, pers. comm.). Among patients who died, 75% (9/12) of deaths were TB-related, and 67% (6/9) occurred within 49 days of TB diagnosis. Among HIV-infected patients, failure or inability to use an effective injectable TB medication was associated with TB-related death.

Some diagnostic and treatment practices contributed to successful outcomes. Among symptomatic patients, initial TB diagnosis was made relatively quickly, within 7 weeks of symptom onset. Nearly three-fourths (73%) of patients were hospitalized; duration was 1 month for non-XDR TB patients and 9 months for XDR TB patients. Extensive DST of first-line and second-line medications was conducted within 2 months of treatment initiation for most patients. At some point during treatment, 61% of patients were receiving a 5-drug regimen. For outpatient care, DOT was used nearly universally, including during home isolation as recommended by national guidelines (*7*). The physicians of most patients with MDR/XDR TB consulted with experts. However, patient management required intensive monitoring and numerous medication changes. Case management services were also intensive; a case manager was assigned to ≈90% of patients.

Deficiencies in practices were identified. Despite CDC recommendations, ≈20% of patients had no documentation of expert consultation. Outcome was unknown for 3% who transferred within and 6% outside the United States and for 2% who were lost to follow-up. Acquisition of any drug resistance during treatment occurred for 21% of patients and was more likely to occur in populations difficult to treat (those who had pre-XDR TB or XDR TB or were homeless), suggesting a need for more vigilant treatment monitoring. There were delays of ≈3 months before patients started a 5-drug regimen. Today, the use of more rapid molecular diagnostic techniques could shorten the time to initiation of an appropriate treatment regimen (*18*). Since September 2009, CDC has offered US sites a molecular-based testing service (www.cdc.gov/tb/topic/laboratory/guide.html).

Because MDR TB treatment lasts >2 years (vs. 6 months for drug-susceptible TB), uses expensive medications, and requires hospitalization for ≈75% of patients (vs. 50% with drug-susceptible TB), it was very costly to treat and manage; average direct cost was $134,000 per MDR TB patient and $430,000 per XDR TB patient. The estimated $17,000 per non-MDR TB patient is 8 and 25 times lower than the costs for MDR TB and XDR TB, respectively. In contrast, lifetime care per HIV-infected patient costs $380,000 (updated to 2010 dollars) (*19*) and lifetime care per breast cancer patient costs $20,000–$100,000 (*20*).

During 2005–2007, the 373 MDR/XDR TB cases cost the US health care system an estimated $53 million; during this time there were ≈41,000 total TB cases. Direct costs for an average XDR TB patient were 3.2 times those for an average MDR TB patient, mostly because of hospitalization costs. Only 20% of XDR TB and 28% of MDR TB patients were managed solely as outpatients. The public sector incurred 80% of the MDR TB costs and 100% of the XDR TB costs.

With health care reform, a substantial proportion of uninsured TB patients are expected to become eligible for Medicaid coverage, which should increase access to health care and early TB diagnosis and decrease TB-associated hospitalizations and deaths. Prevention opportunities for MDR TB are limited. Maintaining the capacity of public health departments and of publicly financed hospitals to act as safety nets (regardless of patient insurance status) to quickly diagnose MDR/XDR TB and isolate and effectively treat the patients will be critical for preventing deaths and transmission of drug-resistant TB organisms. Investment in infection control infrastructure and the capacity to prevent TB among MDR TB patient contacts is also critical; our cost-of-illness estimates did not include these programmatic costs of preventing cases. During the 1979–1994 TB resurgence and MDR TB outbreaks in the United States, New York City renovated hospitals and the Rikers Island prison and treated 20,000 excess TB patients at a cost of ≈$1.7 billion (updated to 2010 dollars) (*21*).

This study had some limitations. Detailed hospitalization records were unavailable for 17 patients. Moreover, documentation of care for incarcerated patients was limited. Follow-up data for all patients were unavailable after 1 year of treatment. Because outpatient care was provided by a mixture of public and private providers, we used average wholesale medication prices to estimate medication costs, which overestimated actual costs to TB clinics that have access to reduced (often one half to one third) medication prices negotiated with pharmaceutical companies. Estimates of out-of-pocket costs were not included.

## Conclusions 

In this population-based sample of MDR/XDR TB patients in the United States, despite the extensive drug resistance found at the time of diagnosis, culture conversion and treatment completion rates were high and mortality rates were low. Records of treatment practices documented near-universal use of DOT. However, these outcomes came at a high cost to the public sector, providing incentives for the United States to prevent MDR/XDR TB. Preventing MDR/XDR TB in the United States will require addressing factors associated with development of drug resistance in countries where foreign-born US patients originate, as well as rapid diagnosis, appropriate regimen selection, robust case management practices, and continued emphasis on DOT in the United States.

Technical Appendix Additional methods, cost analysis, definitions, and additional results with regard to treatment practices, outcomes, and costs of MDR TB and XDR TB in the United States, 2005–2007.
