# Supplementary material for: Treatment Practices, Outcomes, and Costs of Multidrug-Resistant and Extensively Drug-Resistant Tuberculosis, United States, 2005–2007
Source: Emerg Infect Dis. 2014 May;20(5):812–21. doi: 10.3201/eid2005.131037 (PMC4012799; doi:10.3201/eid2005.131037)
Supplement: Technical Appendix — Additional methods, cost analysis, definitions, and additional results with regard to treatment practices, outcomes, and costs of MDR TB and XDR TB in the United States, 2005–2007. [file 13-1037-Techapp-s1.pdf]

Article DOI: DOI: http://dx.doi.org/10.3201/eid2005.131037

Treatment Practices, Outcomes, and Costs of Multidrug-Resistant and Extensively Drug-Resistant Tuberculosis,United States, 2005–2007

Technical Appendix

Contents

1. Additional Methods
    a. Sampling
    b. Data Collection
    c. Data Analysis
2. Cost Analysis
    a. Inpatient Cost Estimation
    b. Outpatient Cost Estimation
    c. Total Direct Cost Estimation
    d. Productivity Loss Estimation
    e. Cost Analyses
3. Definitions
4. Additional Results
    a. Percent of Patients Having Isolates Tested within 30 days of Medications Start and Resistance to Medications
    b. Percentage of Patients on Various Medications at Any Time During Treatment
    c. Median Months to Event
    d. Acquired Resistance Multivariate Model
    e. Death During TB Treatment Multivariate Model A
    f. Death During TB Treatment Multivariate Model B
    g. Inpatient Direct Costs Boxplot
    h. Outpatient Direct Costs Boxplot
    i. Total Direct Costs Boxplot
    j. Productivity Losses Boxplot
    k. Direct Costs and Productivity Losses Boxplot
    l. MDR TB Direct Costs Multivariate Model
    m. MDR TB Direct Costs and Productivity Losses Multivariable Model
    n. Cost Estimation of Drug-susceptible TB Costs

Additional Methods

Sampling

Each participating site identified MDR-TB and XDR-TB patients reported to CDC during 2005-2007. All XDR-TB patients were selected for participation in the study. From the sampling

frame of MDR-TB patients, a simple random sample of 75% was selected from California and New York and of 50% from Texas. In one instance in New York, a randomly selected patient whose records were unavailable was replaced with another randomly selected patient.

Data Collection

Data were abstracted onto standardized data collection forms, one for TB clinic records and one for each hospitalization, which were entered into separate databases. Separate Excel spreadsheets were used for direct entry of abstracted data on specimens, chest radiography/CT exams, and medications. All databases were converted to SAS for analysis.

Data Analysis

From the originally collected variables, we created the following new variables:

- First date of a positive specimen
- First and last date of positive specimen culture
- Sputum-culture-conversion date: the first date (at least 7 days after the date of last sputum-culture positivity) of 2 consecutive negative sputum-culture results, with 3 categories of exceptions:
    - If there were not 2 consecutive negative culture results 7 days after the last positive, used the available date 7 days after (3 patients).
    - If there were 2 consecutive negative culture results, but the 2nd result was more than a month after the first and the first is not quite 7 days from the last positive, use the first even though it is not quite 7 days after (1 patient)
    - If there were at least 2 negative culture results after the last positive, but none met the 7 day criteria, use the last one available (otherwise the patient would have qualified as no culture conversion) (1 patient)
- First date and duration of TB medications
- First date of diagnosis: earliest of report date, first medication, first positive specimen
- First date and duration of symptoms: from various documented symptoms
- First date and duration of respiratory isolation, intensive care unit, mechanical ventilation
- Days hospitalized: sum of days hospitalized over all TB-related hospitalizations
- Any resistance to each medication
- Initial resistance to each medication within 30 days of TB treatment start
- Acquired resistance to any medication: any resistant result after a susceptible result
- Five mutually exclusive categories of drug resistance: INH/RIF/RBT-only, INH/RIF/RBT-plus, First-line, Pre-XDR, XDR
- Milligrams per medication per week per patient: dose*frequency per week*weeks
- Days to culture conversion: date of culture conversion minus date of TB medications start
- Medication duration: days on each individual medication before and after culture conversion
- Effective medication duration: days on each individual medication to which the isolate was never resistant
- Start and duration of a 5-drug regimen: the date the patient started taking at least 5 medications (including an INJ and FQ) to which their TB isolate was never resistant
- Days infectious: number of days 12 weeks prior to first symptom or, if asymptomatic, prior to initial diagnosis through culture conversion or medications stop
- Number of outpatient DOT visits per patient: DOT regimen*frequency per week*weeks

- Total number of chest radiographs and CTs per patient
- Disseminated disease: TB in ≥ 2 noncontiguous sites at any time during treatment
- Died during treatment: if the reason for treatment stop was died, or hospital disposition was died
- History of TB: if there was a history of TB from either clinic or hospital records or patient self-report
- History of latent TB infection: if there was a history of LTBI from either clinic or hospital records or patient self-report
- Homeless: if in a homeless living situation within the year prior to TB treatment or hospitalization, or if homeless at TB diagnosis or during TB treatment
- Prison/jail inmate: if incarcerated within the year prior to TB treatment or hospitalization, or if an inmate at TB diagnosis or during treatment
- Unemployed: if primary occupation within the year prior to TB treatment was "unemployed"
- Uninsured: if no health insurance during TB treatment, separately for clinic and hospitalization
- Smoker: if patient smoked at least once within the year prior to clinic intake or hospitalization
- Long-term-care facility resident: if the patient was a resident of a long-term-care facility at TB diagnosis or within 3 months prior to TB treatment start

The following data analyses were performed:

- Compared the representativeness of study data with National TB Surveillance System (NTSS) data[1]
- Examined homelessness prior to TB, during TB treatment, housing assistance
- Examined unemployment prior to TB, because of TB
- Summarized DST testing by medication, initial resistance, ever resistance, and acquired resistance
- Calculated the median number of DSTs on medications, median number of medications having resistance
- Assessed associations with pre-XDR and XDR combined vs. other MDR patients
- Examined resistance patterns by country of birth, compared with NTSS all-TB data by country of birth
- Identified medications to which resistance was acquired during treatment
- Identified factors associated with any acquired resistance
- Calculated the percentage of patients diagnosed while hospitalized
- Assessed symptoms
- Calculated the median days to initial diagnosis
- Calculated the median months of infectiousness
- Calculated the percentage of patients on each medication at any time during treatment
- Calculated the medication changes per patient, reasons
- Examined whether patients were hospitalized, the number of hospitalizations, and reasons for hospitalization
- Calculated the outpatient DOT use per medication
- Calculated the number of medications taken prior to culture conversion to which the patient's isolate was never resistant

- Calculated the median days from medications start to 5-drug regimen start, 5-drug regimen to culture conversion, any regimen to culture conversion, 5-drug regimen to treatment stop, by drug resistance pattern
- Calculated the median treatment length and by resistance pattern and 5-drug regimen
- Identified factors associated with treatment longer than 2 years after culture conversion
- Assessed culture conversion by resistance pattern and by number of effective medications prior to culture conversion
- Analyzed treatment outcomes by resistance pattern, comparison with US MDR/XDR and U.S. drug-susceptible cases
- Calculated the number of days to TB-related death, by 5-drug regimen and for HIV+ by highly active antiretroviral treatment (HAART)
- Calculated the median days on effective medications before and after culture conversion by resistance pattern
- Identified characteristics associated with death during treatment
- Calculated the median and average number of adverse events by resistance pattern and association with individual and combined medications
- Calculated the median days of inpatient (inpatient days, respiratory isolation) and outpatient care (total outpatient, DOT days, outpatient isolation)
- Measured the number of outpatient procedures: chest radiographs, smears, cultures
- Assessed receipt of expert consultation, associations with expert consultation
- Calculated case management usage types and frequency
- Assessed use of interpreters by non-English speaking patients
- Analyzed impairment during treatment

Cost Analysis: Inpatient Cost Estimation

From total charges per TB-related hospitalization that were collected, inpatient costs were calculated by:

- Converting all charges to 2010 inflation-adjusted dollars using the Consumer Price Index for Medical Care[2]
- Converted charges to costs by multiplying the charges by each hospital's cost-to-charge ratio[3]
    - o If a specific hospital's cost-to-charge ratio was unavailable, we used operating-cost-to-charge ratio average for the state (CA=0.32, NY=0.46, TX=0.40)
- Converted site-specific costs, using Medicare Geographic Adjustment Factors[4] as the cost-of-living adjustment factor, to a US basis for aggregation (CA=1.13, NY=1.08, TX=0.94)
- Added in physician costs for each hospitalization day from the 2009 Physicians' Fee and Coding Guide of $238.50 for the first day of hospitalization at Level 2,[5] converted to a cost by multiplying by the average cost-to-charge ratio for pulmonary diseases 0.502,[6] updated to 2010 dollars using the Medical Care CPI,[2] for a value of $123.81. The remaining hospitalization days were calculated similarly from a 2009 charge of $131 to a cost of $68/day.
- For 17 patients for whom we were not able to obtain detailed hospital data, the length of stay (which was obtained for all patients) was multiplied by an average cost per day ($1419) for hospitalizations for which we did have data

Cost Analysis: Outpatient Cost Estimation

Outpatient costs were estimated as follows:

- Computed the number of outpatient days of treatment
- Estimated outpatient diagnosis costs, using numbers of chest radiographs (2 views $30.97), computed tomographies ($308.99), AFB smears ($7.70), *M.tuberculosis* cultures ($15.48), DSTs ($8.10), and other diagnostic tests, and applied Centers for Medicare and Medicaid Lab Schedule Fees[7]
- Often, DOT visits were not specified as taking place at the clinic or at home, so we estimated that 11% of DOT visits occurred in the clinic (5 days/week for 6 weeks=30 visits, plus 25 visits for the remaining 490 work days of a 2-year period of 520 work days), and the remaining 89% of DOT visits occurred in the patient's home. Costs for a home DOT visit ($32.70) were estimated using the 2001 Physicians' Fee and Coding Guide Medicare Allowable Charge for a home DOT visit by a nurse ($49) [8] and updating it to 2010 dollars using the change in home health care average weekly earnings[2] and converting it to a cost by multiplying by 50.2%, the average cost to charge ratio for pulmonary diagnostic related group (DRG) [6]: (49*(484.58/364.51)*.502)). Costs for clinic DOT visits ($38.63) were estimated using the 2009 Physicians' Fee and Coding Guide average outpatient physician visit charge for an established level 2 patient ($58.67 for Level 2 patient[5]), then updated to 2010 dollars using the CPI/MC, converted to a cost by multiplying by 0.502, plus 15 minutes of a nurse based on the cost of an outpatient DOT visit: (58.67*(388.436/375.613)*.502)+(.25*(49*(484.58/364.51)*.502))).
- Estimated outpatient medication costs by multiplying the total outpatient mg. of each medication by its Red Book average wholesale price per mg.[9] Added therapeutic monitoring costs (CBC $11.14, LFT $11.70), according to the Drug-Resistant Tuberculosis Survival Guide for Clinicians,[10] for those on CYC, AMK, CAP, KAN. Costs per mg by medication were:
    - INH=(0.000517778)
    - RIF=(0.006702778)
    - RBT=(0.102664)
    - RPT=(0.02765)
    - PZA=(0.002381)
    - EMB=(0.004448)
    - SM=(0.00432)
    - CAP=(0.175)
    - KAN=(0.006041667)
    - AMK=(0.06771)
    - CIP=(0.0026254)
    - LEV=(0.0385332)
    - MOX=(0.048954167)
    - GAT=(0.10723)
    - ETH=(0.0168836)
    - PAS=(0.000934417)
    - CYC=(0.024)
    - CLO=(0.2022)
    - LIN=(0.194871667)
    - CLR=(0.008477)

    - AZI=(0.04242)
- Estimated, using Centers for Medicare and Medicaid Lab Schedule Fees,[7] outpatient lab tests for baseline tests (CBC with WBC differential [$11.14], comprehensive metabolic panel $15.14, LFT [$11.70], HIV EIA initial [$19.65] and follow-up Western Blot test [$38.35] if positive, TSH [$24.06] if on CYC or ETH, and 6 drug levels [$21.59] for those on CAP, KAN, AMK, SM, or CYC). Estimated ongoing lab tests, based on the MDR-TB Survival Guide,[10] for those on treatment longer than one week (monthly CBC, monthly LFT, monthly comprehensive metabolic panel for those on CAP, KAN, AMK, SM, monthly audiometry [$34.38] if on AMK, CAP, KAN, SM, monthly visual function screening [$3.06] if on EMB, CLO, RBT, LIN, quarterly TSH for those on CYC, ETH, PAS)
- Estimated case management costs, based on the frequency of each type of case management.
    - Transportation costs were estimated at $20/week in LA, $29/week in NYC, $17.50/week in TX.
    - Housing costs were estimated at $31/day in CA, $37/day in NY, $19/day in TX.[11]
    - Social worker costs were estimated at $23.95 for 2 days consultation. [12]
    - Interpreters were estimated at $21.73/hour [12]
- Calculated outpatient direct costs as the sum of outpatient diagnosis, outpatient DOT, outpatient medication, outpatient lab, and case management costs.

Cost Analysis: Total Direct Cost Estimation

Estimated total direct costs as the sum of outpatient direct costs and inpatient direct costs.

Cost Analysis: Productivity Loss Estimation

Estimated productivity losses as follows:

- Calculated a disability adjustment based on responses to Adverse Events variable on the clinic form, 100% divided into 6 segments (100%, 83%, 67%, 50%, 33%, 17%)
- Estimated inpatient productivity losses as follows: Applied a value of work plus home production, updated to 2010 dollars using the change in average hourly earnings,[13] at $224/day for employed patients, and just home production ($40) for non-employed to each day hospitalized[14]
- Estimated outpatient productivity losses as follows: ½ day for each DOT day, a full day if in outpatient isolation, times the disability adjustment factor for all remaining outpatient days
- Estimated one year of productivity losses for one year after treatment for one person who had documentation, at 50% disability, and discounted it using a 3% social discount rate.
- For TB-related deaths (9/13 deaths), applied a value of remaining lifetime productivity, updated to 2010 dollars using the change in average hourly earnings,[15] based on the age at death[14]
- Estimated total productivity loss as the sum of inpatient productivity loss, outpatient productivity loss, after treatment productivity loss, and productivity loss due to premature death.

Cost Analyses

- Estimated 3 illness severity categories similar to analysis done in the previous TB hospitalization study:
    - Category 1= those who died from TB-related causes
    - Category 2= any comorbidities, pregnancy, disseminated or extensive disease
    - Category 3= all remaining cases
- Calculated average costs by resistance pattern, by illness severity, and for each cost category
- Identified characteristics associated with high direct and societal costs
- Estimated U.S. MDR/XDR direct and direct-plus-productivity-loss costs during 2005-2007 by applying average costs per study patient to the U.S. cohort of cases during the period.
- Assessed who paid the costs by public sector/private sector insurance

Study Definitions
Initial diagnosis: earliest of TB medication start, collection of first positive specimen (AFB-smear, M. tuberculosis culture, PCR, HPLC, or NAA), or report date (date the health department first suspected a patient had TB)

First-line TB Medications: isoniazid (INH), rifampin (RIF), rifabutin (RBT), pyrazinamide (PZA), ethambutol (EMB), streptomycin (SM),
Second-line TB Medications: capreomycin (CAP), kanamycin (KAN), amikacin (AMK), any second-line injectable (INJ), ciprofloxacin (CIP), ofloxacin (OFL), levofloxacin (LEV), moxifloxacin (MOX), gatifloxacin (GAT), any fluoroquinolone (FQ), ethionamide (ETH), para-aminosalicylic acid (PAS), cycloserine (CYC),
Third-line TB Medications: clofazimine (CLO), linezolid (LIN), clarithromycin (CLR), azithromycin (AZI)

MDR TB: resistance to at least INH and RIF

Initial resistance: any resistance found on the initial TB isolate cultured within 30 days of specimen collection in the U.S.

Acquired resistance: any resistance found after a documented prior susceptible culture

Resistance patterns, mutually exclusive:
- INH/RIF/RBT-only
- INH/RIF/RBT-plus other resistance to any of the following: PZA, EMB, SM, PAS, ETH, or CYC (excluding total first-line resistance)
- Total First-line: resistance to INH, RIF, PZA, and EMB
- Pre-XDR: MDR TB plus resistance to a FQ or an INJ
- XDR: MDR TB plus resistance to both a FQ and a INJ

5-drug-regimen: an INJ, a FQ, and ≥ 3 other medications to which M. tuberculosis was never resistant

Infectious period: began 12 weeks before earliest of first symptom, first medication, first positive specimen collection, or report date; ended at culture conversion, or, if no culture conversion, censored at medications stop

Date of culture conversion: After the last positive-sputum-culture, the first date (≥7 days after last positive) of 2 consecutive negative-sputum-culture results

Disseminated disease: ≥ 2 non-contiguous sites of disease

Extensive disease: incapacitating, preventing normal everyday activities

Moderate disease: sufficiently discomforting to interfere with normal everyday activities

Minimal disease: causing minimal discomfort, easily tolerated, not interfering with everyday activities

Additional Results

Percent of Patients Having Isolates Tested within 30 days of Medications Start and Resistance to Medications, N=134

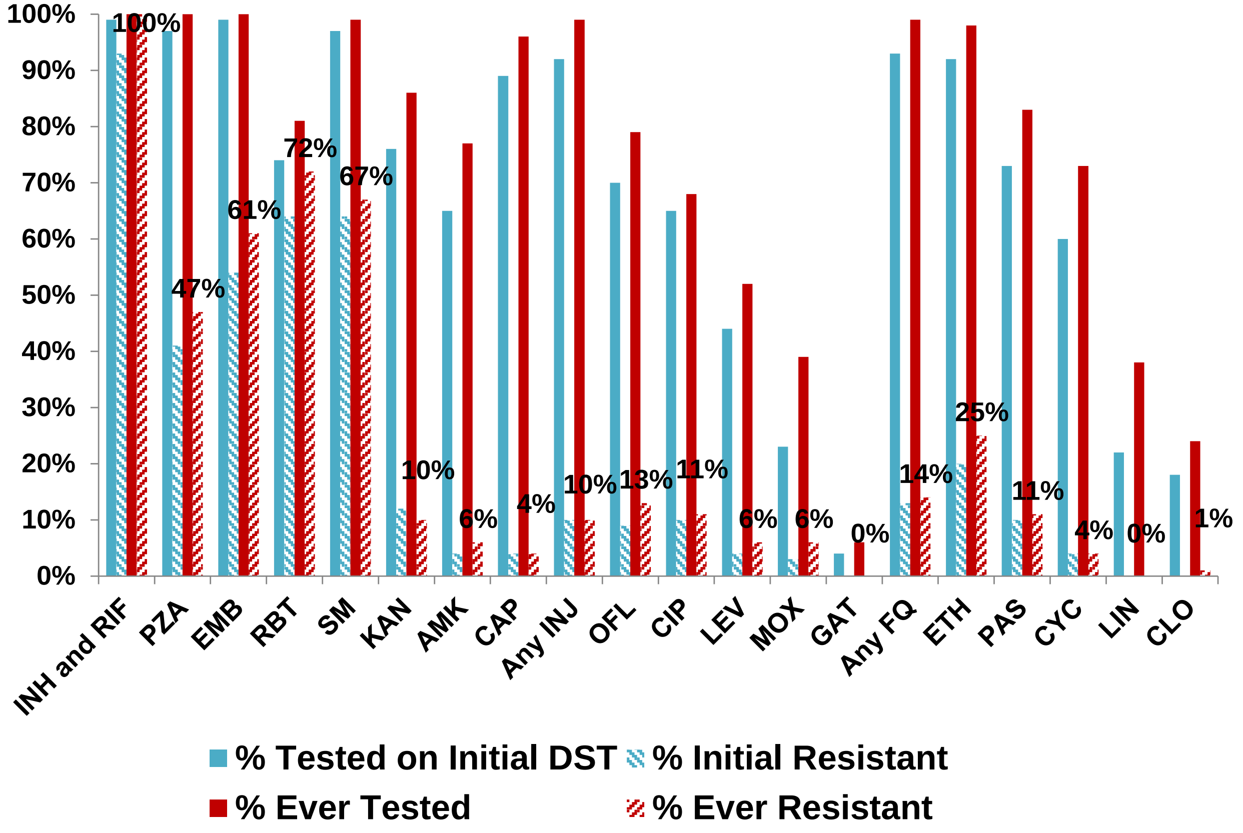

Percentage of Patients on Various Medications at Any Time During Treatment (N= 134 patients)*

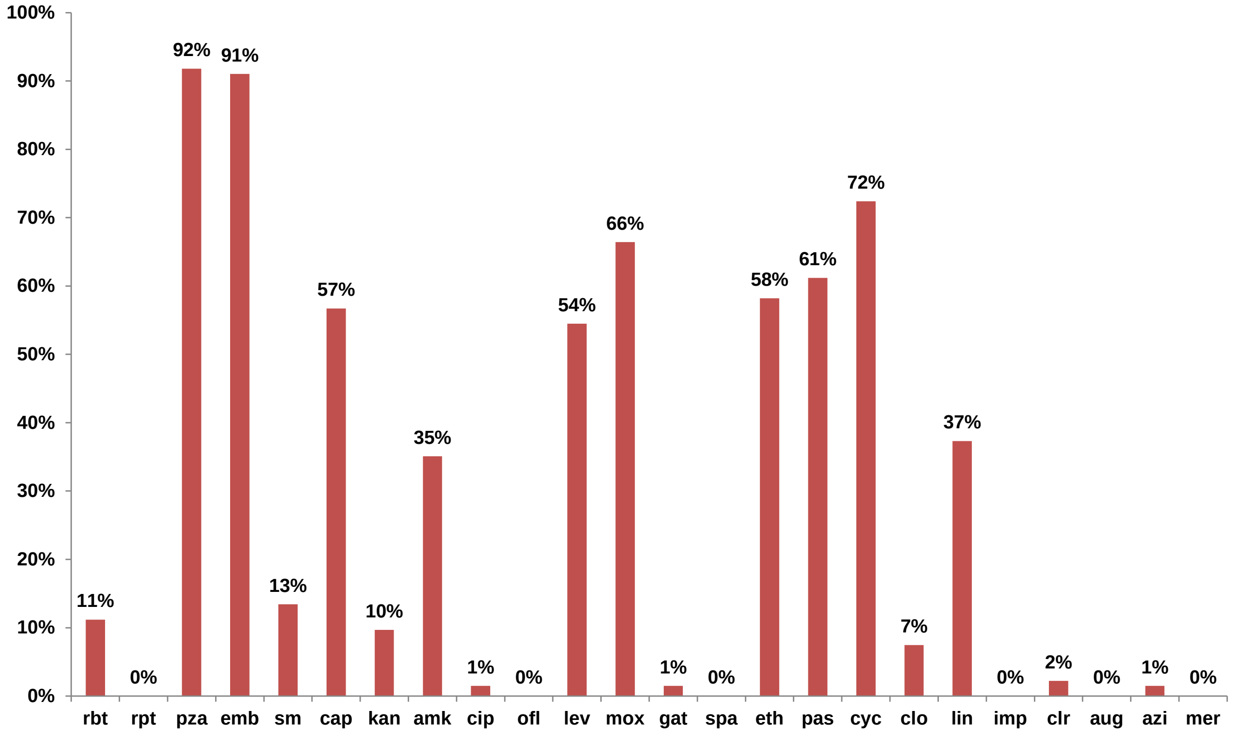

* 1 patient was diagnosed at death and was not started on medications

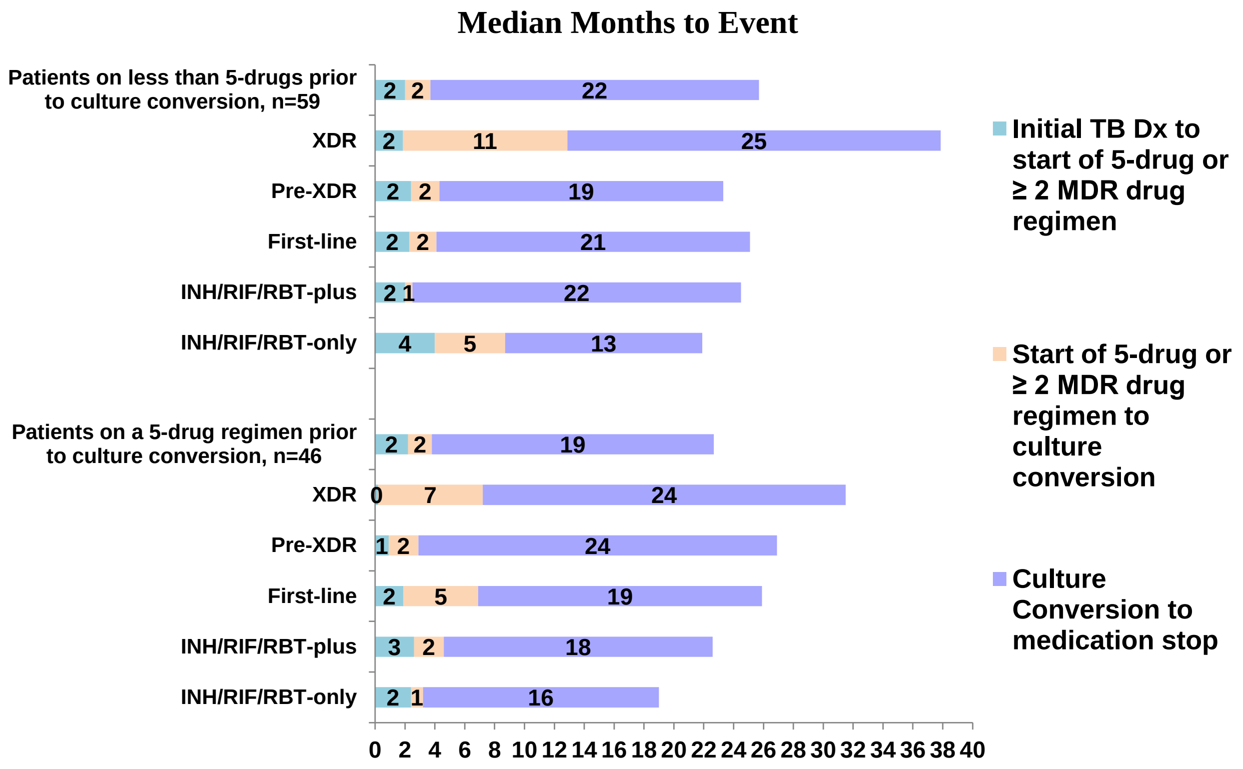
Median Months to Event
Patients on less than 5-drugs prior to culture conversion, n=59
XDR
Pre-XDR
First-line
INH/RIF/RBT-plus
INH/RIF/RBT-only
Patients on a 5-drug regimen prior to culture conversion, n=46
XDR
Pre-XDR
First-line
INH/RIF/RBT-plus
INH/RIF/RBT-only
2 2 22
2 11 25
2 2 19
2 2 21
2 1 22
4 5 13
2 2 19
0 7 24
1 2 24
2 5 19
3 2 18
2 1 16
0 2 4 6 8 10 12 14 16 18 20 22 24 26 28 30 32 34 36 38 40
Initial TB Dx to start of 5-drug or ≥ 2 MDR drug regimen
Start of 5-drug or ≥ 2 MDR drug regimen to culture conversion
Culture Conversion to medication stop

Acquired Resistance Multivariate Models

Variable	Initial Odds Ratio Estimate	Initial 95% Confidence Lower Limit	Initial 95% Confidence Upper Limit	Initial Pr > ChiSq	Final Odds Ratio Estimate	Final 95% Confidence Lower Limit	Final 95% Confidence Upper Limit	Final Pr > ChiSq
Age 65 or over	<0.001	<0.001	>999.999	0.973				
**Black race**	33.19	0.80	>999.999	0.065	**4.07**	**1.08**	**15.37**	**0.039**
**Recent homelessness**	18.76	0.93	377.71	0.056	**5.81**	**1.17**	**28.86**	**0.031**
**Pre-XDR or XDR TB**	8.78	2.31	33.42	0.001	**5.15**	**1.86**	**14.21**	**0.002**
AFB smear positive	5.34	0.86	33.22	0.072				
Age 25-44 years	4.83	0.65	36.03	0.124				
Hispanic ethnicity	4.83	0.11	216.20	0.417				
≥ 4 effective medications	3.46	0.41	29.47	0.256				
Age 45-64 years	3.41	0.39	30.21	0.271				
Asian race	3.35	0.07	151.32	0.534				
Disseminated TB disease	2.28	0.21	24.20	0.495				
Foreign born	2.10	0.15	28.64	0.577				
Recent cigarette smoker	1.96	0.41	9.31	0.397				
Recent excess alcohol use	0.99	0.13	7.65	0.988				
TB clinic outpatient management	0.98	0.18	5.35	0.980				
Recent unemployment	0.97	0.24	3.93	0.964				
Cavitary disease	0.77	0.21	2.84	0.694				
Diabetes	0.76	0.16	3.70	0.732				
History of TB disease	0.44	0.12	1.59	0.208				
HIV infection	0.39	0.03	4.92	0.463				
Recent injecting-drug or non-injecting-drug use	0.15	0.00	5.08	0.291				

Model Fit Statistics

Criterion	Initial Model Intercept and Covariates	Final Model Intercept and Covariates
**AIC**	136.392	116.619
**SC**	198.964	**127.996**
**-2 Log L**	92.392	108.619

Death During TB Treatment Multivariate Model A

**Death During Treatment Model A**		**Initial**	**Initial**			**Final**	**Final**	
	**Initial**	**95%**	**95%**		**Final**	**95%**	**95%**	
	**Odds Ratio**	**Confidence**	**Confidence**	**Initial**	**Odds Ratio**	**Confidence**	**Confidence**	**Final**
**Variable**	**Estimate**	**Lower Limit**	**Upper Limit**	**Pr > ChiSq**	**Estimate**	**Lower Limit**	**Upper Limit**	**Pr > ChiSq**
**Recent cigarette smoker**	>999.999	2.60	>999.999	0.023	**6.39**	**1.04**	**39.41**	**0.046**
Age 45-64 years	>999.999	<0.001	>999.999	0.924				
Age 25-44 years	>999.999	<0.001	>999.999	0.947				
**Age 65 or over**	>999.999	<0.001	>999.999	0.890	**20.21**	**2.26**	**180.98**	**0.007**
Recent excess alcohol use	690.52	0.16	>999.999	0.126				
Foreign born	303.05	0.10	>999.999	0.164				
**HIV infection**	99.82	0.09	>999.999	0.201	**6.31**	**1.05**	**37.73**	**0.044**
TB clinic outpatient management	17.19	0.13	>999.999	0.251				
Hispanic ethnicity	17.14	0.00	>999.999	0.567				
Black race	14.02	0.00	>999.999	0.565				
Asian race	6.00	0.00	>999.999	0.682				
Disseminated TB disease	2.23	0.01	852.99	0.792				
Recent unemployment	0.83	0.05	14.21	0.900				
Recent homelessness	0.48	0.00	82.71	0.777				
Pre-XDR or XDR TB	0.36	0.01	16.56	0.598				
History of TB disease	0.26	0.01	6.02	0.401				
Diabetes	0.04	<0.001	12.94	0.268				
Male	0.02	<0.001	2.40	0.107				
Recent injecting-drug or non-injecting-drug use	0.01	<0.001	12.50	0.186				
Pregnant	0.00	<0.001	>999.999	0.990				
Correctional inmate	<0.001	<0.001	>999.999	0.971				
**Model Fit Statistics**								
	**Initial Model**	**Final Model**						
	**Intercept**	**Intercept**						
	**and**	**and**						
**Criterion**	**Covariates**	**Covariates**						
**AIC**	72.127	56.51						
**SC**	135.88	**68.101**						
**-2 Log L**	28.127	48.51						

Death During TB Treatment Multivariate Model B

Death During Treatment Model B<br>Variable	Initial Odds Ratio Estimate	Initial Pr > ChiSq	Final Odds Ratio Estimate	Final 95% Confidence Lower Limit	Final 95% Confidence Upper Limit	Final Pr > ChiSq
**Age ≥65**	0.00	0.9774	**61.926**	**3.099**	**>999.999**	**0.0069**
Age ≥65*Excess alcohol	1.00	.				
Age ≥65*Black race	1.00	.				
Age ≥65*efffluorom	1.00	.				
Age ≥65*effective INJ med	>1000	0.9983				
Age ≥65*Foreign born	1.00	.				
Age ≥65*HIV-infection	1.00	.				
Age ≥65*Homeless	1.00	.				
Age ≥65*Hispanic	1.00	.				
Age ≥65*IDU or NIDU	1.00	.				
Age ≥65*Smoker	>1000	0.9984				
Age ≥65*TB clinic	>1000	0.9791				
Age ≥65*TB history	0.00	0.9774				
Excess alcohol	0.00	0.9986				
Excess alcohol*effective FQ med	1.00	.				
Excess alcohol*effective INJ med	1.00	.				
Excess alcohol*Smoker	>1000	0.9986				
Excess alcohol*TB clinic	1.00	.				
Excess alcohol*TB history	1.00	.				
Black race	0.02	0.9959				
Black race*Excess alcohol	1.00	.				
Black race*effective FQ med	1.00	.				
Black race*effective INJ med	0.00	0.9996				
Black race*Foreign born	>1000	0.9994				
Black race*HIV-infection	>1000	0.9996				
Black race*Homeless	0.00	0.9968				
Black race*IDU or NIDU	1.00	.				
Black race*Smoker	>1000	0.9994				
Black race*TB clinic	1.32	0.9992				
Black race*TB history	1.00	.				
effective FQ med	0.00	0.9792				
**effective INJ med**	0.00	0.9629	**0.017**	**0.002**	**0.176**	**0.0006**
effective INJ med*effective FQ med	>1000	0.976				
Foreign born	1.26	0.9997				
Foreign born*Excess alcohol	0.05	0.9964				
Foreign born*effective FQ med	1.00	.				
Foreign born*effective INJ med	1.00	.				
Foreign born*HIV-infection	>1000	0.9996				
Foreign born*Homeless	1.00	.				
Foreign born*IDU or NIDU	>1000	0.9983				
Foreign born*Smoker	1.71	0.9991				
Foreign born*TB clinic	4.60	0.998				
Foreign born*TB history	1.00	.				
HIV-infection	0.00	0.9995				
HIV-infection*Excess alcohol	>1000	0.9983				
HIV-infection*effective FQ med	1.00	.				
HIV-infection*effective INJ med	1.00	.				
HIV-infection*Homeless	>1000	0.9972				
HIV-infection*IDU or NIDU	0.00	0.9976				
HIV-infection*Smoker	0.05	0.9965				
HIV-infection*TB clinic	1.00	.				
HIV-infection*TB history	>1000	0.9982				
Homeless	>1000	0.9969				
Homeless*Excess alcohol	>1000	0.9982				
Homeless*effective FQ med	1.00	.				
Homeless*effective INJ med	1.00	.				
Homeless*IDU or NIDU	1.00	.				
Homeless*Smoker	>1000	0.9996				
Homeless*TB clinic	1.00	.				
Homeless*TB history	1.00	.				
Hispanic	6.79	0.9978				
Hispanic*Excess alcohol	>1000	0.9984				
Hispanic*Black race	1.00	.				
Hispanic*effective FQ med	0.01	0.9905				
Hispanic*effective INJ med	71.72	0.9907				
Hispanic*Foreign born	0.14	0.9975				
Hispanic*HIV-infection	56.90	0.9965				
Hispanic*Homeless	0.00	0.9969				
Hispanic*IDU or NIDU	0.00	0.999				
Hispanic*Smoker	0.52	0.9978				
Hispanic*TB clinic	1.39	0.9984				
Hispanic*TB history	2.03	0.9956				
IDU or NIDU	>1000	0.9991				
IDU or NIDU*Excess alcohol	0.00	0.9977				
IDU or NIDU*effective FQ med	1.00	.				
IDU or NIDU*effective INJ med	1.00	.				
IDU or NIDU*Smoker	1.00	.				
IDU or NIDU*TB clinic	1.00	.				
IDU or NIDU*TB history	1.00	.				
male	0.00	0.9755				
male*Age ≥65	0.00	0.9994				
male*Excess alcohol	10.85	0.996				
male*Black race	1.68	0.9985				
male*effective FQ med	>1000	0.9742				
male*effective INJ med	42.97	0.9916				
male*Foreign born	0.46	0.9989				
male*HIV-infection	65.29	0.9963				
male*Homeless	0.00	0.998				
male*Hispanic	2.69	0.9937				
male*IDU or NIDU	>1000	0.9982				
male*Smoker	0.46	0.9984				
male*TB clinic	1.17	0.9992				
male*TB history	2.74	0.9934				
**Smoker**	1.73	0.999	**19.387**	**2.727**	**137.817**	**0.0031**
Smoker*effective FQ med	1.00	.				
Smoker*effective INJ med	1.00	.				
Smoker*TB clinic	1.12	0.9997				
Smoker*TB history	0.53	0.9979				
TB clinic	0.22	0.9981				
TB clinic*efffluorome	0.14	0.9911				
TB clinic*effective INJ med	5.50	0.9957				
TB history	0.07	0.9948				
TB history*effective FQ med	0.27	0.9939				
TB history*effective INJ med	36.59	0.9924				
TB history*TB clinic	1.05	0.9997				

Model Fit Statistics<br>Criterion	Initial Model Intercept and Covariates	Final Model Intercept and Covariates
**AIC**	140.006	40.932
**SC**	342.855	**52.524**
**-2 Log L**	0.006	32.932

Inpatient Direct Costs

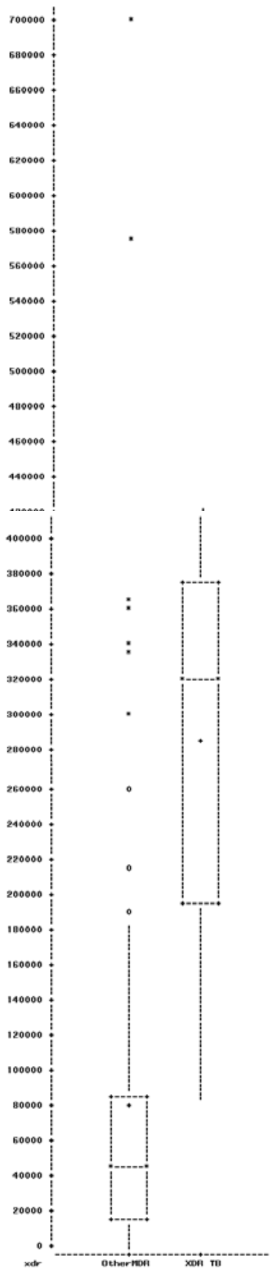

Standard error of the mean:

Other MDR	12,200
XDR	70,829

Outpatient Direct Costs

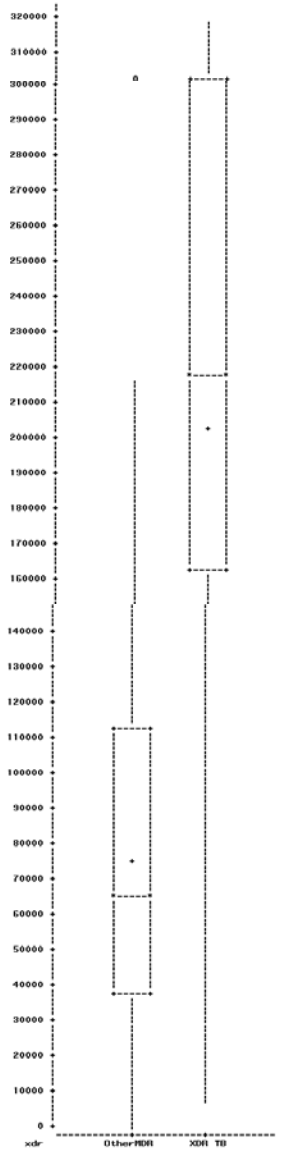

Standard error of the mean:

Other MDR	4,991
XDR	56,374

Total Direct Costs

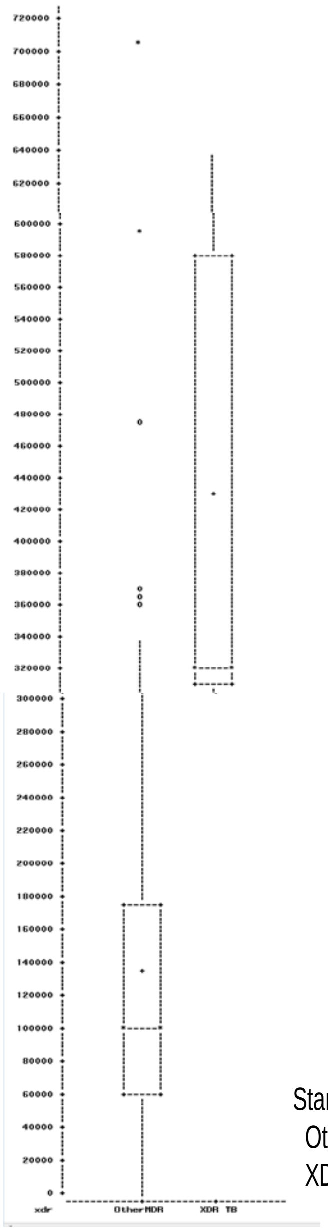

Standard error of the mean:

Other MDR	9,683
XDR	73,109

Productivity Losses

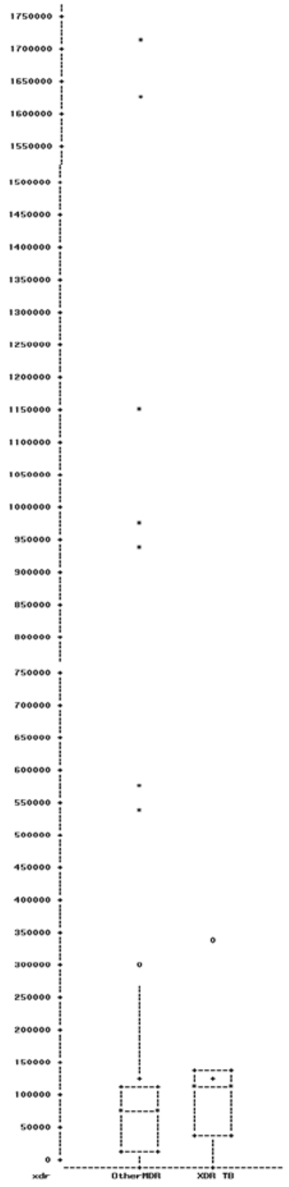

Standard error of the mean:

Other MDR	22,520
XDR	59,594

Direct Costs and Productivity Losses

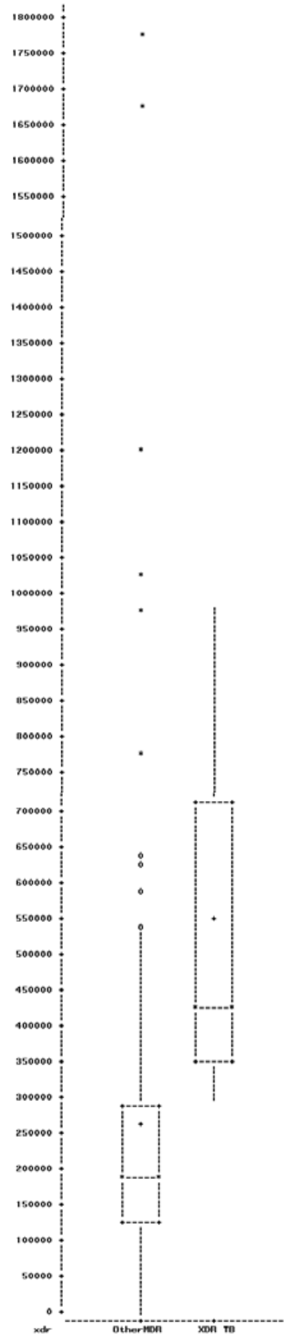

Standard error of the mean:

Other MDR	23,212
XDR	127,707

MDR-TB Direct Costs Multivariate Model

Variable	Initial Parameter Estimate	Initial Pr > F	Final Parameter Estimate	Final Standard Error	Final F Value	Final Pr > F
**Intercept**	29,310	0.559	**80,807**	**11,139**	**52.620**	**<.0001**
**XDR TB**	242,811	<.0001	**219,438**	**42,494**	**26.670**	**<.0001**
**Long-term care facility resident**	208,619	0.003	**182,545**	**64,022**	**8.130**	**0.005**
**Recent non-injecting-drug use**	119,934	0.065	**112,203**	**42,984**	**6.810**	**0.010**
Black race	86,395	0.054				
**HIV infection**	58,745	0.135	**69,368**	**33,234**	**4.360**	**0.039**
Asian race	54,566	0.238				
Recent homelessness	44,402	0.314				
**Public insurance**	43,185	0.053	**57,221**	**17,052**	**11.260**	**0.001**
Hispanic ethnicity	41,324	0.371				
Total firstline resistance	28,626	0.196				
Foreign born	11,142	0.747				
Male	9,828	0.606				
Pre-XDR TB	9,263	0.712				
Diabetes	3,845	0.889				
**No. of days hospitalized**	451	<.0001	**426**	**60**	**50.540**	**<.0001**
**Recent injecting-drug use**	-324,397	0.000	**-315,716**	**70,089**	**20.290**	**<.0001**
**Recent excess alcohol use**	-78,044	0.033	**-74,682**	**33,009**	**5.120**	**0.026**
Age 45-64 years	-34,156	0.240				
Age 65 or over	-30,517	0.487				
Correctional inmate	-24,708	0.632				
Recent unemployment	-14,145	0.535				
Age 25-44 years	-14,492	0.568				
Native Hawaiian/Other Pacific Islander	-1,829	0.986				
**Model Statistics**	**Adj R-Square=.52**			**Adj R-Square = 0.55**		
**Analysis of Variance**						
**Source**	**Degrees of freedom**		**Sum of Squares**	**Mean Square**	**F Value**	**Pr > F**
**Model**	**8**		**1,160,877,000,000**	**145,109,600,000**	**18.84**	**<.0001**
**Error**	**111**		**854,750,200,000**	**7,700,452,335**		
**Corrected**	**119**		**2,015,627,000,000**			

MDR-TB Direct Costs and Productivity Losses
Multivariate Model

Variable	Initial Parameter Estimate	Initial Pr > F	Final Parameter Estimate	Final Standard Error	Final F Value	Final Pr > F
**Intercept**	41,006	0.688	**175,161**	**27,059**	**41.900**	**<.0001**
**Died**	837,849	<.0001	**810,082**	**82,887**	**95.520**	**<.0001**
**XDR TB**	322,504	0.001	**315,817**	**91,413**	**11.940**	**0.001**
**Recent non-injecting-drug use**	188,405	0.158	**240,819**	**93,512**	**6.630**	**0.011**
Hispanic ethnicity	151,102	0.112				
**HIV infection**	144,532	0.084	**151,514**	**71,251**	**4.520**	**0.036**
Asian race	137,699	0.154				
Native Hawaiian/Other Pacific Islander	104,329	0.628				
**Diabetes**	83,578	0.140	**123,628**	**47,944**	**6.650**	**0.011**
**Male**	63,478	0.104	**65,679**	**35,964**	**3.340**	**0.071**
Recent injecting-drug use	91,371	0.588				
Black race	52,318	0.566				
Public insurance	57,079	0.209				
Total firstline resistance	24,606	0.584				
Age 25-44 years	37,381	0.470				
**No. of days hospitalized**	525	0.000	**424**	**128**	**11.020**	**0.001**
**Age 65 or over**	-379,300	0.000	**-428,817**	**80,756**	**28.200**	**<.0001**
**Long-term care facility resident**	-357,057	0.020	**-280,788**	**137,546**	**4.170**	**0.044**
**Correctional inmate**	-158,124	0.134	**-180,258**	**91,947**	**3.840**	**0.053**
**Recent excess alcohol use**	-149,285	0.049	**-179,858**	**67,536**	**7.090**	**0.009**
**Age 45-64 years**	-67,604	0.253	**-123,917**	**39,748**	**9.720**	**0.002**
Recent unemployment	-68,354	0.146				
Pre-XDR TB	-50,252	0.328				
Foreign born	-35,118	0.627				
Recent homelessness	5,003	0.956				
**Model Statistics**	**Adj R-Square=.64**			**Adj R-Square = 0.66**		
**Analysis of Variance**						

Source	Degrees of freedom		Sum of Squares	Mean Square	F Value	Pr > F
**Model**	**12**		**7,719,364,000,000**	**643,280,300,000**	**19.91**	**<.0001**
**Error**	**107**		**3,456,381,000,000**	**32,302,623,791**		
**Corrected**	**119**		**11,175,740,000,000**			

Estimate of the Average Cost of Non-MDR TB, 2010 U.S. Dollars									
Site	Avg hospitalization length	1998$ avg cost per patient hospitalized*	2010$ avg cost per patient hospitalized**	2010$ hospital physician cost***	2010$ avg total hospitalization cost per patient	Weight	2010$ weighted avg cost per pt. hospitalized	2010$ outpatient cost per patient****	2010$ avg cost per TB patient*****
1	17	$11,987	$19,233	$1,290	$20,523	6.2	$127,485	$3,419	
2	37	$22,518	$36,130	$2,605	$38,735	12.2	$473,212	$3,419	
3	35	$12,231	$19,623	$2,474	$22,097	7.1	$157,255	$3,419	
4	15	$13,631	$21,871	$1,159	$23,030	10.8	$248,245	$3,419	
5	22	$15,114	$24,250	$1,619	$25,869	5.5	$141,508	$3,419	
6	20	$17,651	$28,320	$1,487	$29,808	13.6	$404,616	$3,419	
7	24	$17,009	$27,290	$1,751	$29,040	14.1	$409,356	$3,419	
8	36	$22,316	$35,806	$2,540	$38,345	17.9	$685,538	$3,419	
9	23	$10,438	$16,746	$1,685	$18,431	13.8	$254,907	$3,419	
10	16	$11,970	$19,205	$1,224	$20,429	14.7	$299,492	$3,419	
Total						115.8	$3,201,614		
**Average**							**$27,640**	**$3,419**	**$16,963**

*** From reference 16: 49% of TB patients were hospitalized; included MDR TB costs for 13/733 hospitalized patients**
**** Converted from 1998 dollars to 2010 dollars using the consumer price index for medical care (388.436/242.1) reference 2**
***** Average of lower and upper physician fees (reference 5) for one day of initial hospital care ($206,$271)=$238,**
and remaining days at Level 2 subsequent care ($113,$149)=$131,
converted to a cost using the cost-to-charge ratio for the respiratory Diagnostic Related Group (reference 6), multiplied by 0.502
****** From reference 17, $2985 from Nashville, TN, adjusted to a US basis using the Census Bureau Cost of Living Index 87.3 (reference 18)**
******* (Weighted average hospitalization cost X 49% of patients hospitalized)+average outpatient cost**

References

1. Centers for Disease Control and Prevention. Online TB Information System (OTIS 2009, wonder.cdc.gov/TB-v2009.html), accessed March 2012.

2. Bureau of Labor Statistics. Consumer Price Index—all urban consumers, medical care. Series ID CUUR0000SAM. [cited February 21, 2012] http://data.bls.gov/cgi-bin/srgate

3. Centers for Medicare and Medicaid Services. 2005 Operating cost to charge ratios (OPCCR). FY2005 Impact file (inpatient prospective payment system) [cited May 12, 2010].http://www.cms.gov/Medicare/Medicare-Fee-for-Service-Payment/PCPricer/index.html?redirect=/pcpricer

4. Centers for Medicare and Medicaid Services. 2005 Medicare geographic adjustment factors. [cited May 12, 2010] http://www.cms.gov/Research-Statistics-Data-and-Systems/Statistics-Trends-and-Reports/Medicare-Geographic-Variation/GV_PUF.html

5. Mag Mutual Healthcare Solutions, Inc. 2009 Physicians' fee & coding guide. Duluth (GA). 2008.

6. The Diagnosis-Related Group Handbook. comparative clinical and financial standards. Ernst & Young LLP. Washington, DC. 1998:21.

7. Centers for Medicare and Medicaid Services. Clinical laboratory fee schedule [cited February 9, 2012]. www.cms.gov/Medicare/Medicare-Fee-For-Service-Payment/Clinicallabfeesched/index.html

8. Health Care Consultants of America, Inc. 2001 Physicians Fee and Coding Guide. Augusta, Georgia. 2002.

9. Red Book Online. Thompson and Reuters Red Book. Average wholesale prices [cited 2012 Mar]. www.micromedexsolutions.com

10. Francis J. Curry National Tuberculosis Center and California Department of Public Health. Drug-Resistant Tuberculosis: A Survival Guide for Clinicians. 2nd ed.; 2008.

11. US Department of Housing and Urban Development. Fair Market Rents. http://www.huduser.org/portal/datasets/fmr.html

12. US. Department of Labor. Wages, earnings, and benefits [cited 2012 Feb]. http://www.dol.gov/dol/topic/statistics/wagesearnings.htm

13. Bureau of Labor Statistics. Average Hourly Earnings of Production and Nonsupervisory Employees; Series CEU0500000008.

14. Grosse SD. Appendix I: productivity loss tables. In Haddix AC. Teutsch SM, Corso PS, editors. Prevention effectiveness: a guide to decision analysis and economic evaluation. 2nd ed. Oxford University Press; New York, New York. 2003:245-257.

15. Taylor Z, Marks SM, Rios Burrows NM, Weis SE, Stricof RL, Miller B. Causes and Costs of hospitalization of tuberculosis patients in the United States. Int J Tuberc Lung Dis. 2000;4:931–9. PubMed

16. Shepardson D, Marks SM, Chesson H, Kerrigan A, Holland DP, Scott N, et al. Cost-effectiveness of a 12-dose regimen for treating latent tuberculosis infection in the United States. Int J Tuberc Lung Dis. 2013;17:1531–7. PubMed http://dx.doi.org/10.5588/ijtld.13.0423

17. ACCRA Cost of Living Index. Annual Average 2010 via U.S. Census Bureau http://www.census.gov/compendia/statab/cats/prices/consumer_price_indexes_cost_of_living_index.html accessed 02/05/2014.
